# Validation of a novel EC and PPD-based decision tree model for tuberculosis screening in Tibetan adolescent students

**DOI:** 10.3389/fmed.2025.1671278

**Published:** 2025-10-13

**Authors:** Wenying Hong, Yuan Xu, Lu Wen, Yao Zhou, Chunjun Huang

**Affiliations:** ^1^Department of Pharmacy, Chengdu Wenjiang District People’s Hospital, Chengdu, Sichuan, China; ^2^Seda County People's Hospital, Ganzi Tibetan Autonomous Prefecture, Sichuan, China; ^3^Department of Nursing, Chengdu Wenjiang District People’s Hospital, Chengdu, Sichuan, China

**Keywords:** tuberculosis, recombinant *Mycobacterium tuberculosis* fusion protein, tuberculin skin test, combined diagnostic decision tree, high-altitude regions

## Abstract

**Objective:**

To evaluate the utility and effectiveness of the recombinant *Mycobacterium tuberculosis* fusion protein (EC) skin test for tuberculosis (TB) screening among student populations in high-altitude regions and to provide evidence-based recommendations for optimizing epidemic control strategies.

**Methods:**

A total of 1,047 primary and secondary school students in Seda County were enrolled. Both the tuberculin skin test (TST/PPD) and EC skin test were administered to all participants. Data analysis was performed using R 4.3.0 and Python 12.0 statistical software. Descriptive analyses included skewed continuous data expressed as median (Q₁, Q₃) and analyzed using the Kruskal-Wallis test, while categorical data were presented as n (%) and analyzed using Chi-square or Fisher’s exact tests. Model construction and performance evaluation were implemented in Python, utilizing packages such as graphviz, matplotlib, and scikit-plot for visualization and metrics calculation.

**Results:**

Based on expert consensus, participants were stratified into three groups: BCG vaccination (*n* = 29, 2.77%), uninfected (*n* = 975, 93.12%), and at least latent infection (including both latent TB infection and active TB, *n* = 43, 4.11%). The PPD test showed significant intergroup differences (*p* < 0.001), with AUC values of 0.98 (BCG vaccination), 0.92 (uninfected), and 0.83 (at least latent infection), and an overall Kappa coefficient of 0.59. The EC test demonstrated perfect performance in identifying latent infections (precision, recall, F1-score, and AUC = 1.00) but failed to distinguish BCG-vaccinated individuals (all metrics = 0). A decision tree model combining EC + PPD demonstrated perfect classification performance on the current dataset, achieving accuracy, recall, and AUC values of 1.00 across all classifications, with a micro-average AUC of 1.00 and a Kappa coefficient of 1.00.

**Conclusion:**

While the EC skin test exhibits 100% sensitivity for latent TB infection, it cannot differentiate between persistent post-vaccination positivity and true uninfected status. The EC + PPD decision tree model synergistically optimizes multi-dimensional metrics, enabling high-sensitivity detection of latent infections and precise exclusion of false positives, thereby improving overall diagnostic performance. This integrated approach could improve TB screening accuracy in high-altitude student populations, inform targeted public health interventions, and warrants further validation. While this study was conducted in a high-altitude region, the combined EC + PPD approach warrants evaluation in other settings with high BCG vaccination rates.

## 1 Introduction

Tuberculosis (TB), caused by *Mycobacterium tuberculosis* (MTB), is a chronic infectious disease and one of the top 10 causes of death worldwide. As a global leader in TB control, China has achieved the TB-related targets of the United Nations Millennium Development Goals 5 years ahead of schedule (1), contributing valuable experience to global TB prevention efforts. However, the current prevention and control situation remains challenging. The 2024 WHO Global Tuberculosis Report indicates that China had an estimated 741,000 new TB cases in 2023, accounting for 6.8% of global cases. Although this represents a decrease from 2022, China still ranks third in the world for TB incidence (2).

With dense populations, schools are high-risk settings for TB outbreaks. Given that children and adolescents— the primary population in schools— still bear an unacceptably high TB burden, school-based TB prevention has become a key focus of China’s TB control efforts (3, 4). The World Health Organization estimates that 1.2 million children and young adolescents developed TB in 2022, with a staggering 50% of cases going undetected by public health systems (5). The root of this diagnostic crisis lies in the unique pathophysiology of TB in the young. Children often present with non-specific symptoms and paucibacillary disease, rendering pathogen-based tests like smear microscopy insensitive. Concurrently, their evolving immune systems result in variable performance of host-based immune assays. Consequently, the failure to diagnose leads to a failure to treat, accounting for 96% of TB deaths in this age group (5). Therefore, the development and validation of novel diagnostic tests that can overcome these hurdles—such as those utilizing non-sputum samples or novel antigen targets—are paramount to closing the case detection gap and reducing mortality. In this context, early detection of latent TB infections is critical for preventing TB transmission within schools.

Rapid detection of TB infection (including both active TB and latent TB infection) is essential for TB eradication. However, latent cases lack clinical manifestations, and most active TB cases are difficult to identify in early stages, when bacteriological tests have low positive detection rates. Therefore, indirect testing methods are needed to support clinical TB diagnosis. The World Health Organization (WHO) recognizes two commercialized techniques for TB infection detection: the tuberculin skin test (TST) and interferon-gamma release assays (IGRAs) (2). The tuberculin skin test (TST) is a traditional detection method known for its simplicity and low cost, but it has low specificity in populations vaccinated with BCG or infected with non-tuberculous mycobacteria (NTM). Interferon-gamma release assays (IGRAs) use mycobacterial protein peptides (including ESAT-6, CFP-10, and TB7.7) to stimulate effector lymphocytes to secrete IFN-*γ*, which is then detected and quantified to determine the presence of MTB-specific cellular immune responses (6). These tests are less affected by BCG vaccination or NTM infection but are more expensive and require laboratory support.

Currently, in addition to TST and IGRAs, China has independently developed a new diagnostic product called the recombinant *Mycobacterium tuberculosis* fusion protein (EC). This protein induces specific delayed-type hypersensitivity (DTH) reactions to distinguish MTB infection status, offering high sensitivity, strong specificity, and simple operation. It can be used for diagnosing latent TB infection (LTBI) and culture-negative pulmonary TB (7). However, there is limited research on EC’s application in TB screening in high-altitude regions. This study conducted EC and TST skin tests among students in a high-altitude area to evaluate the value of EC testing for TB screening in such settings.

## 2 Materials and methods

### 2.1 Study population

A total of 1,047 students from primary and secondary schools in Seda County, a high-altitude region, were enrolled for tuberculosis screening. All participants provided written informed consent and underwent both EC skin test and TST.

### 2.2 Diagnostic reagents

Recombinant *Mycobacterium tuberculosis* fusion protein (EC) 50 U/1.0 mL/vial/box (Zhifei Longcom Biopharmaceutical Co., Ltd., Anhui); Tuberculin purified protein derivative (TB-PPD) 50 IU:1 mL/vial×5/box (Beijing Xiangrui Biological Products Co., Ltd.).

### 2.3 Testing procedures

All 1,047 students underwent simultaneous bilateral arm testing with TB-PPD and EC. Using the Mantoux method, 0.1 mL (5 U) of EC was first injected intradermally on the volar aspect of the left forearm. After 5 min of observation with no abnormalities, 0.1 mL (5 IU) of TB-PPD was injected into the right forearm. Injection sites were examined 48–72 h post-injection. For EC, the larger of erythema or induration was recorded, while for TB-PPD, only induration was measured. The transverse and longitudinal diameters (mm) of reactions were documented, with an average diameter ≥5 mm considered positive. To facilitate direct result comparison under identical conditions, this study administered the EC and PPD tests simultaneously on the contralateral forearms of participants. Close monitoring throughout the process revealed no cross-reactivity or systemic adverse reactions—findings that validate the safety of this concurrent testing approach in our study cohort.

### 2.4 Diagnostic criteria

Reference: “expert consensus on clinical application of recombinant *Mycobacterium tuberculosis* fusion protein (EC).” any cases showing vesiculation, necrosis or lymphangitis were classified as strong positive reactions. Students under 15 years old with any positive screening result (PPD or EC) and all students above 15 years old received chest DR examinations (with CT and molecular biological tests when necessary). All screened subjects underwent sputum smear microscopy and mycobacterial culture. The final diagnostic classification was based on a comprehensive assessment integrating skin test results, radiographic findings (DR/CT), and microbiological confirmation (sputum smear and culture), in accordance with the expert consensus (7). Specifically: BCG vaccination group: PPD positive AND EC negative AND normal chest imaging AND negative microbiological results. Uninfected group: PPD negative AND EC negative AND normal chest imaging (if performed). At least latent infection group (including LTBI and TB): defined by a positive EC test result. Those with normal chest imaging AND negative microbiological results are classified as latent tuberculosis infection (LTBI). Those with abnormal chest imaging AND positive microbiological results are classified as active tuberculosis (TB). A definitive diagnosis of active TB required a positive mycobacterial culture (7).

### 2.5 Statistical analysis

#### 2.5.1 Algorithm description

A Classification and Regression Tree (CART) algorithm was used to build the diagnostic model from the EC and PPD test results. The model was developed using a balanced dataset (n = 279 per group) to mitigate the impact of imbalanced group sizes. To optimize model performance and prevent overfitting, key parameters were tuned, and the complexity of the tree was intentionally limited (e.g., by restricting its maximum depth). The model’s generalizability was assessed using cross-validation.

#### 2.5.2 Statistical methods

R 4.3.0 and Python 12.0 were used for statistical analysis. Descriptive analysis was performed using R: skewed continuous data were expressed as median (Q₁, Q₃) and analyzed using Kruskal-Wallis test; categorical data were presented as n (%) and analyzed using χ^2^ or Fisher’s exact tests, with *p* < 0.05 considered statistically significant. Python was used for model construction. Visualization and performance evaluation included: decision tree diagrams using graphviz package; ROC curves and AUC calculation using matplotlib and scikit-plot; radar charts using matplotlib to comprehensively display model metrics; and learning curves to evaluate model fit, accuracy and confidence interval variability. Comprehensive metrics (F1-score, recall, precision, accuracy and Kappa coefficient) were calculated to thoroughly assess diagnostic performance.

## 3 Results

### 3.1 Sample characteristics and overall screening results

**Figure 1:**
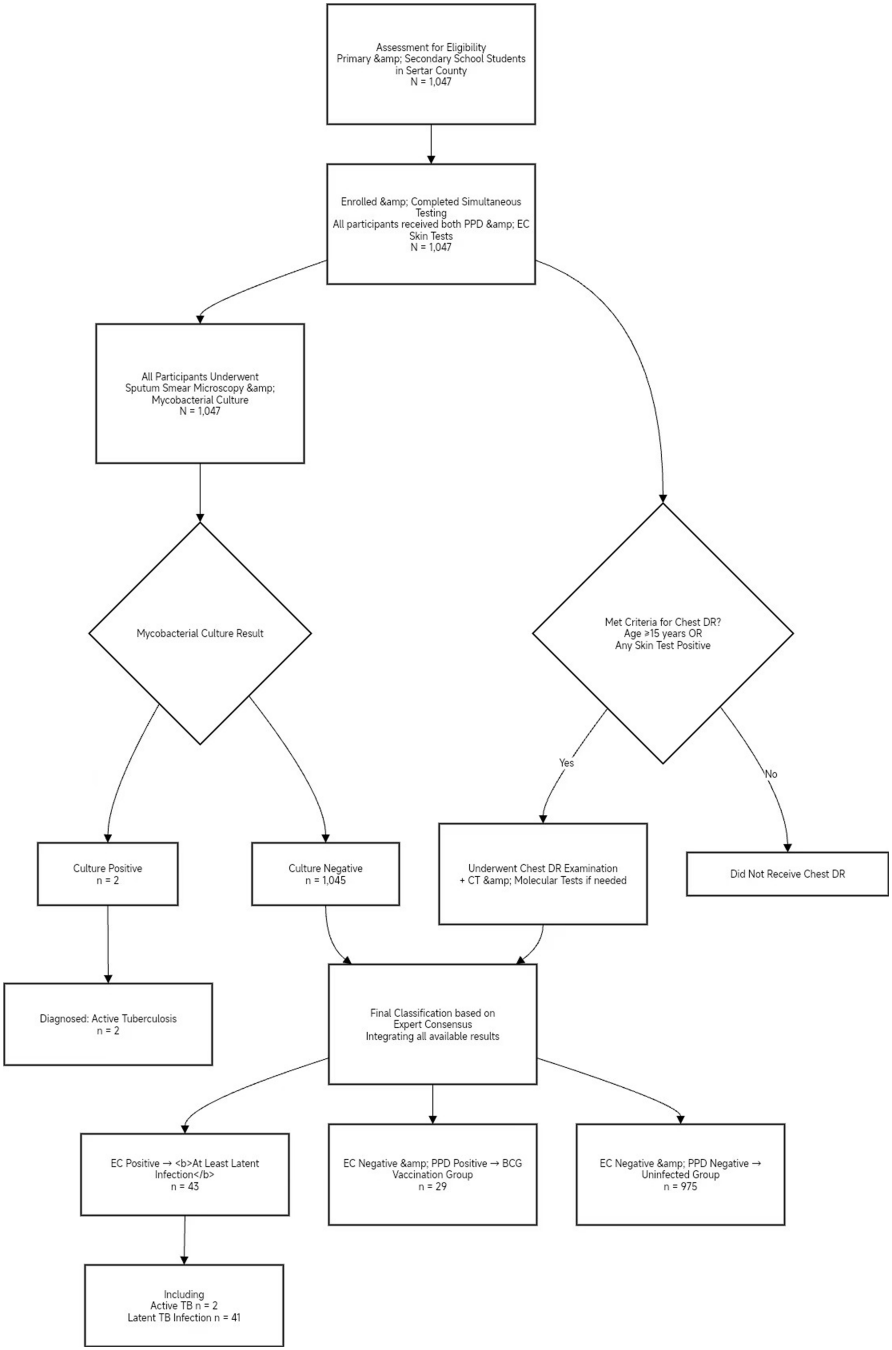
Participant screening, evaluation and classification flowchart. A total of 1,047 primary and secondary school students from Sertar County were enrolled.All participants underwent simultaneous tuberculin skin testing (TST/PPD) and recombinant Mycobacterium tuberculosis fusion protein (EC) testing, as well as microbiological culture (the gold standard for active TB diagnosis). Chest radiography (DR) was performed selectively for students under 15 years with any positive skin test result and for all students aged 15 years or older. Final classification was based on expert consensus criteria integrating all available diagnostic results. The “At least latent infection” group (n = 43) includes individuals with latent tuberculosis infection (n = 41) and those with active, culture-confirmed tuberculosis (n = 2). Notably, both active TB cases exhibited EC-positive/PPD-negative skin test results. Abbreviations: PPD, purified protein derivative; EC, recombinant *Mycobacterium tuberculosis* fusion protein; DR, digital radiography; TB, tuberculosis.

The participant screening, evaluation, and classification workflow is summarized in Figure 1. This study included 1,047 adolescent TB screening participants from high-altitude regions. All subjects underwent both EC and PPD skin tests and were categorized into three groups according to EC expert consensus: BCG vaccination group (*n* = 29, 2.77%; persistent positivity due to BCG vaccination without infection), uninfected group (*n* = 975, 93.12%; no evidence of TB infection), and at least latent infection group (*n* = 43, 4.11%; with latent or active TB infection).

The median ages of the three groups were 12.0, 13.0, and 13.0 years respectively, with no statistically significant differences (H = 3.82, *p* = 0.148). Gender distribution (male 49.09%, female 50.91%) was also balanced across groups (χ^2^ = 0.44, *p* = 0.802).

TB-PPD testing showed 96.55% positivity in BCG vaccination group (58.62% moderate positivity), 100% negativity in uninfected group, and 72.09% positivity in at least latent infection group (44.19% moderate, 11.63% strong positivity), with significant intergroup differences (*p* < 0.001), indicating PPD’s effectiveness in identifying uninfected individuals but difficulty in distinguishing BCG vaccination effects from latent infection. EC testing showed 0% positivity in both BCG vaccination and uninfected groups, but 100% positivity in at least latent infection group, with statistically significant differences in positivity rates (*p* < 0.001), demonstrating EC’s complete exclusion of BCG vaccination interference and accurate identification of latent TB infection (Table 1).

**Table 1:** Baseline characteristics of different groups.

Variables	Total (*n* = 1,047)	BCG vaccination (*n* = 29)	Non-infected (*n* = 975)	At least latent infection (*n* = 43)	Statistic	*p*
Age, M (Q₁, Q₃)	13.00 (12.00, 13.00)	12.00 (7.00, 13.00)	13.00 (12.00, 13.00)	13.00 (12.00, 13.50)	H = 3.82	0.148
Sex, n(%)					χ^2^ = 0.44	0.802
Male	514 (49.09)	15 (51.72)	476 (48.82)	23 (53.49)		
Female	533 (50.91)	14 (48.28)	499 (51.18)	20 (46.51)		
PPD test result, n(%)					−	<0.001
+++	8 (0.76)	3 (10.34)	0 (0.00)	5 (11.63)		
++	36 (3.44)	17 (58.62)	0 (0.00)	19 (44.19)		
++	15 (1.43)	8 (27.59)	0 (0.00)	7 (16.28)		
−	988 (94.36)	1 (3.45)	975 (100.00)	12 (27.91)		
EC test result, n(%)					−	<0.001
Positive	43 (4.11)	0 (0.00)	0 (0.00)	43 (100.00)		
Negative	1,004 (95.89)	29 (100.00)	975 (100.00)	0 (0.00)		

H, Kruskal-wallis test, χ^2^, Chi-square test, −, Fisher exact; M, Median, Q₁, 1st Quartile, Q₃, 3rd Quartile. In the PPD test results, “+++” represents strongly positive, “++” represents moderately positive, “+” represents generally positive, and “-” represents negative.

All 1,047 screened subjects underwent microbiological culture, the gold standard for the diagnosis of active tuberculosis. Active pulmonary tuberculosis was confirmed in only 2 subjects (0.19%). Notably, both culture-positive cases were identified from the subgroup of 72 individuals (6.9% of the total cohort) who tested positive on either skin test, and critically, both were positive for the EC test but negative for the PPD test. This finding indicates that skin testing defined a higher-risk subgroup (comprising 6.9% of the population) for targeted evaluation. Within this subgroup, the positive predictive value (PPV) of a positive skin test for confirming active tuberculosis was 2.8% (2/72). Moreover, the specific pattern of EC-positive/PPD-negative in both active TB cases suggests a potential advantage of the EC test in identifying active disease in this population.

### 3.2 Diagnostic performance of testing methods

#### 3.2.1 PPD test performance

**Figure 2:**
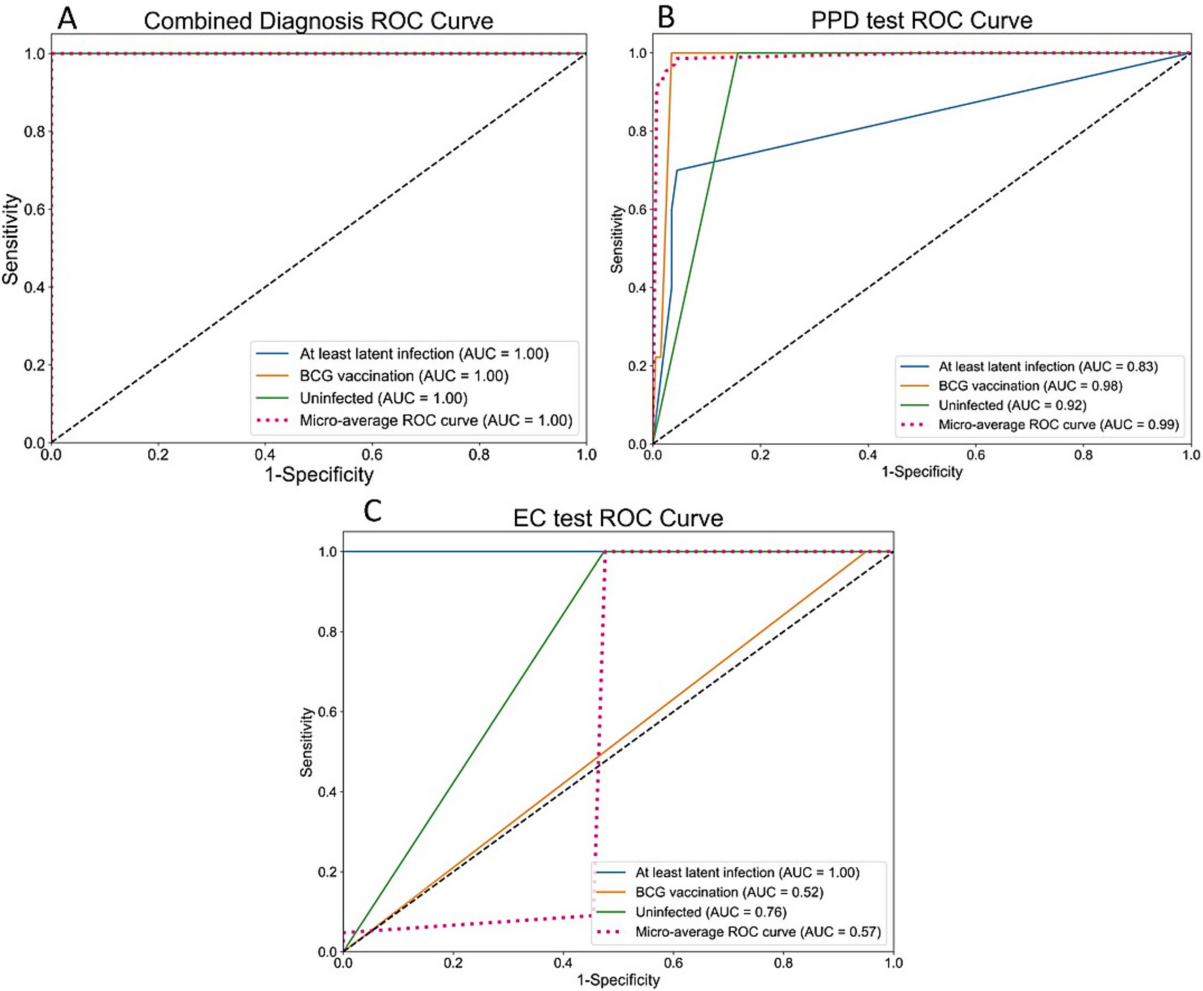
Receiver operating characteristic (ROC) curves evaluating the diagnostic performance. **(A)** The combined EC + PPD decision tree model achieved perfect discrimination (AUC = 1.00) among all three groups. **(B)** The PPD test showed variable performance, with high accuracy in identifying BCG-vaccinated (AUC = 0.98) and uninfected (AUC = 0.92) individuals, but lower performance in identifying infection (AUC = 0.83). **(C)** EC test perfectly identified latent infection (AUC = 1.00) but failed to distinguish BCG-vaccinated individuals.

PPD skin test showed good performance for identifying uninfected individuals (precision = 0.99, recall = 0.99), but only 0.40 precision and 0.57 recall for BCG vaccination group, and 0 for both precision and recall in at least latent infection group (Table 2). ROC curves (Figure 2B) showed AUC values of 0.98 (BCG vaccination), 0.92 (uninfected), and 0.83 (at least latent infection), with a Micro-average Kappa coefficient of 0.59, indicating moderate agreement with gold standard. While PPD effectively excluded uninfected individuals, it showed significant limitations in distinguishing BCG vaccination responses from latent infection.

**Table 2:** Comparison of diagnostic performance for individual categories and kappa coefficients.

Model	Diagnostic Category	Accuracy	Precision	Recall	F1	AUC	Kappa
Combined diagnosis	BCG vaccination	1.00	1.00	1.00	1.00	1.00	1.00
PPD test	BCG vaccination	1.00	0.40	0.57	0.99	0.98	0.59
EC test	BCG vaccination	0.00	0.00	0.00	0.52	0.52	0.81
Combined diagnosis	Uninfected	1.00	1.00	1.00	1.00	1.00	1.00
PPD test	Uninfected	1.00	0.99	0.99	0.88	0.92	0.59
EC test	Uninfected	1.00	0.98	0.99	0.85	0.76	0.81
Combined diagnosis	At least latent infection	1.00	1.00	1.00	1.00	1.00	1.00
PPD test	At least latent infection	0.00	0.00	0.00	0.82	0.83	0.59
EC test	At least latent infection	1.00	1.00	1.00	1.00	1.00	0.81
Combined diagnosis	Micro-average	1.00	1.00	1.00	1.00	1.00	1.00
PPD test	Micro-average	0.96	0.96	0.96	0.99	0.99	0.59
EC test	Micro-average	0.98	0.98	0.98	0.99	0.57	0.81

AUC, Area under the curve. Kappa represents the consistency with the gold standard. Micro-average: A summary metric that calculates the overall performance by counting the total true positives, false negatives, and false positives across all classes. It is useful for evaluating the model’s overall performance in multi-class tasks, especially when class distribution is unbalanced.

#### 3.2.2 EC test performance

EC skin test demonstrated perfect performance for identifying latent infections (precision = 1.00, recall = 1.00, F1-score = 1.00, AUC = 1.00; Figure 2C), but all performance metrics were 0 for BCG vaccination group (Table 2), indicating its inability to distinguish BCG-vaccinated individuals from truly uninfected ones. The EC test yielded a Micro-average AUC of 0.57 and a Kappa coefficient of 0.81. This disparity arises because the Micro-average AUC is heavily penalized by the model’s complete failure to classify the BCG-vaccinated group (AUC = 0.52), while the Kappa coefficient benefits from its perfect performance in identifying the uninfected and latent infection groups. (Table 2; Figure 2).

### 3.3 Construction and performance of combined diagnostic model

**Figure 3:**
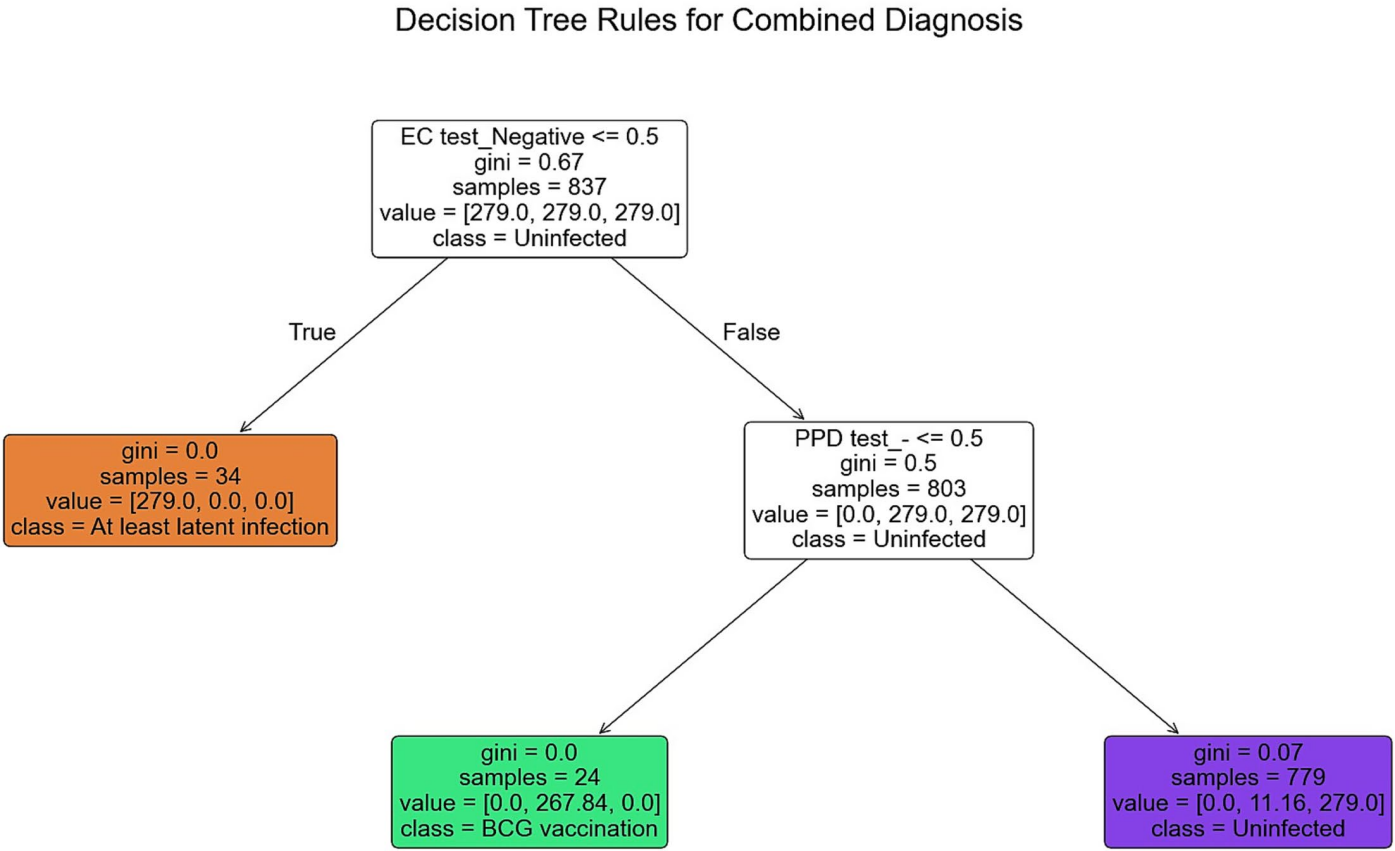
Decision tree model for TB infection classification. The tree uses EC result as the primary split (EC-positive classified as ‘At least Latent infection’) and PPD result for secondary split of EC-negative individuals (PPD-positive classified as ‘BCG-vaccinated’, PPD-negative as ‘Uninfected’).

**Figure 4:**
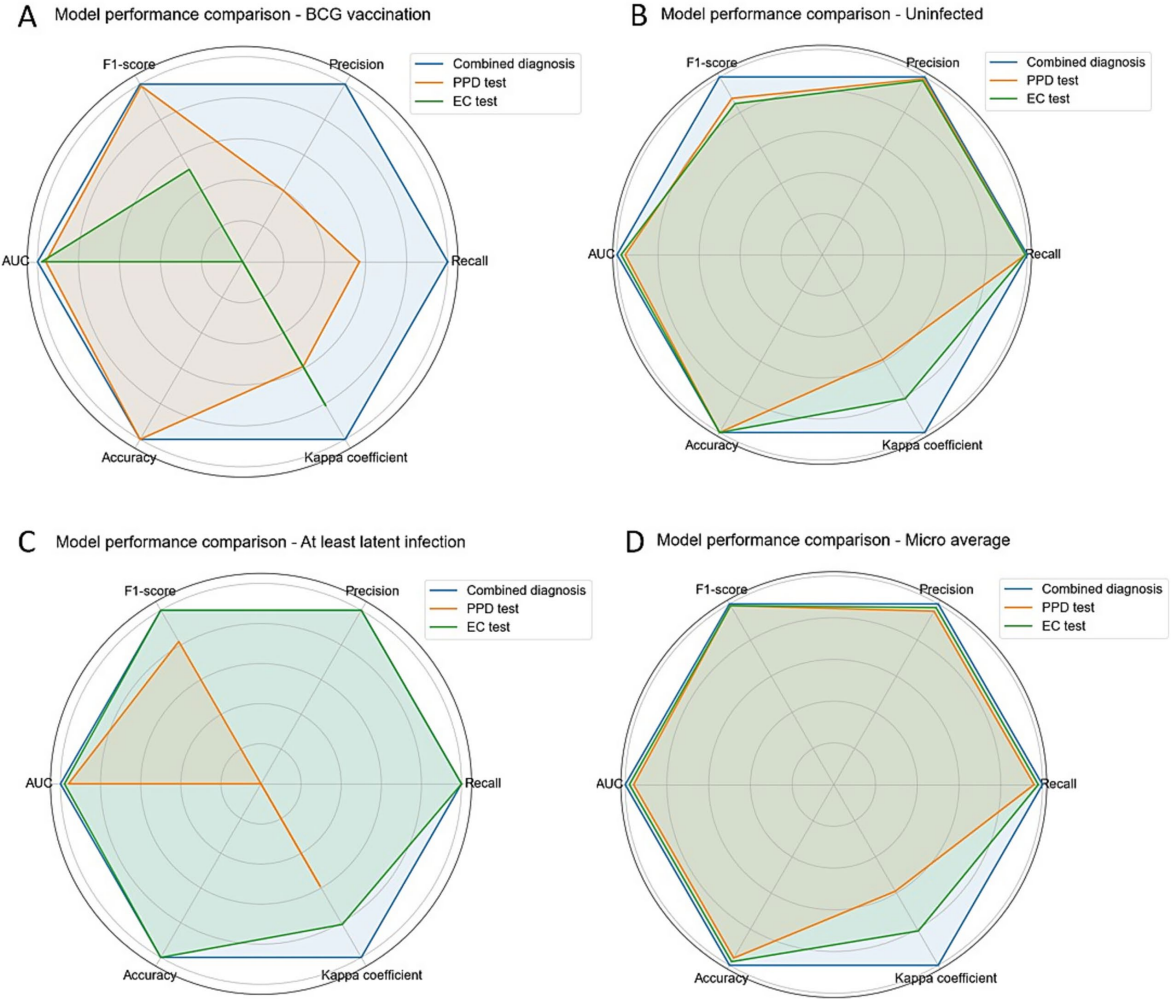
Radar charts visualizing diagnostic performance metrics. **(A–D)** Represent BCG-vaccinated, uninfected, at least latent infection, and micro-average results, respectively. The combined EC + PPD model (blue) demonstrates superior and balanced performance across all metrics (Precision, Recall, F1-Score, AUC, Accuracy) compared to the individual EC (green) or PPD (orange) tests.

In high-altitude regions, widespread BCG vaccination leads to 57% false-positive rate in PPD testing, significantly compromising screening specificity. While EC testing shows 100% sensitivity for latent TB infection, it cannot differentiate between post-vaccination persistent positivity and true non-infection (AUC = 0.52). To overcome these limitations, we integrated EC and PPD results to develop a concise and efficient combined diagnostic decision tree model. Using CART decision tree algorithm on 837 samples (balanced to *n* = 279 per group) with GridSearchCV optimization, we determined optimal parameters: max_depth = 3, min_samples_leaf = 1, min_samples_split = 2. The final decision tree had depth of 2 layers with 5 nodes (1 root, 2 decision, 2 leaf), using Gini impurity as splitting criterion. Primary splitting used “EC test result” (Gini gain = 0.67), classifying EC-positive cases as “at least latent infection” (*n* = 34, Gini = 0). Secondary splitting of EC-negative samples used “PPD result”: PPD-positive as “BCG vaccination” (*n* = 24, Gini = 0) and PPD-negative as “uninfected” (*n* = 779, Gini = 0.07). The model’s “EC-first then PPD” approach initially assumed all samples as “uninfected,” then progressively classified them based on test results (Figure 3). Compared to individual methods, achieved an accuracy, recall, and AUC of 1.00 for all three classifications on the present dataset, with Micro-average AUC = 1.00 and Kappa coefficient = 1.00 (Table 2). Radar charts (Figure 4) visually demonstrated the combined model’s superior performance across all dimensions.

### 3.4 Model stability and generalizability validation

**Figure 5:**
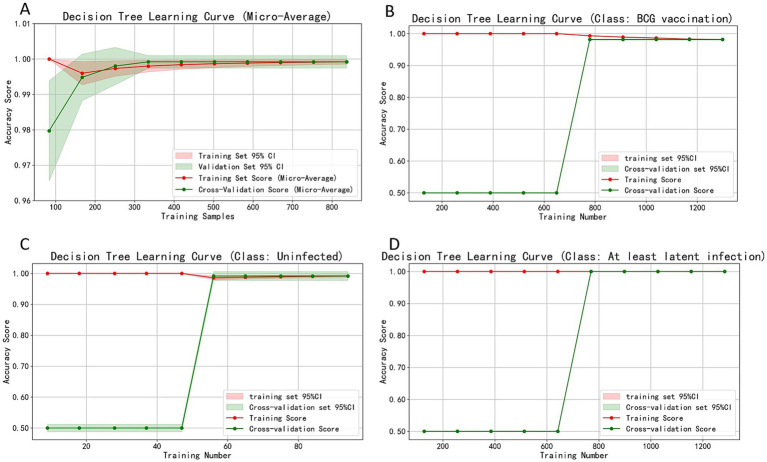
Learning curves assessing model stability and generalizability. **(A)** Micro-average accuracy. **(B–D)** Accuracy for the BCG-vaccinated, Uninfected, and At least latent infection groups, respectively. The convergence of training and cross-validation accuracy curves at high performance levels (0.98–1.00) with increasing sample size indicates the model has low bias and variance, confirming its robustness.

Learning curve analysis using 837 balanced samples (*n* = 279 per group; Figure 5) showed training accuracy rapidly reached 0.99–1.00 after ~150 samples, with cross-validation stabilizing at 150–200 samples. Difference between training and validation narrowed with increasing samples, stabilizing at 0.98–1.00 accuracy at 800 samples (slope≈0). For all three classifications, training accuracy consistently maintained at 1.00, while cross-validation accuracy rose rapidly after inflection points (650, 47, and 650 samples respectively), finally stabilizing at 0.98–1.00. With training/validation accuracy fluctuations <0.01 and 95% CI width <0.03 at maximum sample size, the model demonstrated both low bias (high fit) and low variance (strong generalizability). Appropriate tree depth limits and leaf node sample settings ensured ideal bias-variance balance. The combined EC + PPD decision tree model reached optimal fitting state at 150–650 samples and maintained highly consistent accuracy beyond 800 samples, demonstrating both efficiency and robustness for large-scale screening in high-altitude regions with excellent reproducibility.

## 4 Discussion

Our study adds to the growing body of evidence for recombinant protein skin tests. The high performance of the EC test corroborates findings from a recent systematic review which reported that novel skin tests, including Diaskintest® (Generium, Russian Federation) and C-Tb (Serum Institute of India), demonstrate performance comparable to established tests for LTBI screening (8). These findings collectively demonstrate the utility of this new test class, particularly in BCG-vaccinated populations.

TB is an infectious disease caused by *Mycobacterium tuberculosis* that can affect multiple organs, with pulmonary infection being the most common. It remains a significant global health threat due to its insidious onset, long incubation period, prolonged treatment course, and the potential for drug resistance (2, 9). China is one of the countries with a high burden of tuberculosis. Since the founding of the People’s Republic, advancements in public health and improvements in living standards have led to a marked decline in the incidence and mortality rates of TB. However, China is still one of the 30 high-burden countries for TB globally, with approximately 900,000 new cases annually, ranking third in the world (10). Geographically, the distribution of TB in China is uneven, with the western regions, particularly the plateau pastoral areas, showing a significantly higher disease burden. For example, Seda County, a typical plateau pastoral region, faces a particularly severe tuberculosis situation (11). Additionally, occupational and ethnic factors also influence TB distribution, with farmers, herders, and Tibetans showing a significantly higher incidence compared to other groups (12, 13). These characteristics pose challenges for screening strategies, necessitating the development of diagnostic tools with high sensitivity and specificity.

The diagnosis of active tuberculosis (TB) remains challenging due to the limited sensitivity of tests like sputum smear microscopy, which has a positivity rate of only approximately 30% (14, 15). Consequently, many cases are diagnosed based on clinical presentation and imaging, which can lead to delays and misdiagnosis (16, 17). Given these limitations in active case finding, the accurate detection of latent TB infection (LTBI) becomes even more critical for preventing disease progression and transmission. In this context, our study evaluated the recombinant *Mycobacterium tuberculosis* fusion protein (EC) skin test for LTBI screening in a high-altitude area. The EC skin test showed high sensitivity and specificity in identifying latent infections in our cohort, but fails to classify BCG-vaccinated individuals (overall performance = 0.00). This is because EC includes antigens such as ESAT-6 and CFP-10, which are absent in BCG and most non-tuberculous mycobacteria, thus effectively preventing cross-reactivity (18). However, it is theoretically important to note that these antigens are also present in a limited number of NTM species (e.g., *M. kansasii*, *M. marinum*). Nevertheless, as evidenced by the literature (19), NTM are not a significant cause of false-positive skin test results in high TB burden regions like ours. Consequently, the high specificity of the EC test is maintained, and a positive result in this specific population is overwhelmingly indicative of *M. tuberculosis* complex infection, warranting clinical follow-up. In contrast, while the TB-PPD test has a high accuracy rate of 99% for detecting non-infected individuals, it has a false-positive rate of 57% in the BCG-vaccinated group. This is primarily attributed to cross-reactivity due to prior BCG vaccination.

A key finding is that the EC test, due to its high specificity, completely eliminates interference from BCG vaccination, a major drawback of the PPD test. While the EC test itself cannot identify whether a person has been vaccinated with BCG (as this is not its purpose), the combined EC + PPD algorithm successfully distinguishes the BCG-vaccinated group. This is clinically crucial not for identifying vaccination history per se, but for accurately classifying individuals and avoiding the misclassification of BCG responses as latent infection, thereby preventing unnecessary treatment and anxiety.

To address the limitations inherent in individual diagnostic methods for tuberculosis (TB), this study introduces an innovative approach: a “EC first, then PPD” combined Classification and Regression Tree (CART) decision tree model specifically tailored for screening populations in high-altitude regions. This model overcomes the shortcomings of traditional single-test approaches by incorporating two complementary diagnostic methods—the recombinant *Mycobacterium tuberculosis* fusion protein (EC) skin test and the purified protein derivative (PPD) test—in a sequential manner. By combining these tests, the model enhances diagnostic accuracy, particularly in populations that face unique environmental and genetic factors, such as those found in high-altitude Tibetan regions. The optimization of this combined model was conducted using a grid search method on a sample of 837 balanced individuals (*n* = 279 per group). Through this process, the model’s optimal parameters were determined, including a maximum tree depth (max_depth) of 3, a minimum number of samples per leaf (min_samples_leaf) of 1, and a minimum number of samples required to split a node (min_samples_split) of 2. These settings ensure that the model remains simple and interpretable, while still maintaining high diagnostic performance. The decision tree structure is carefully designed to first eliminate all EC-positive samples, regardless of whether the infection is latent or active, with a Gini gain of 0.67, indicating a high discriminatory power for identifying infected individuals. Subsequently, the model differentiates between those who are EC-negative but PPD-positive (likely due to BCG vaccination, resulting in false positives) and those who are both EC-negative and PPD-negative (indicating a non-infected status), with Gini values of 0 for BCG false positives and 0.07 for non-infected individuals. These Gini values reflect the purity and accuracy of the decision-making process at each node, aligning with known immunological mechanisms of tuberculosis infection and BCG vaccination response.

The combined “EC first, then PPD” decision tree model demonstrated high diagnostic performance on our data, achieving an accuracy, recall, and AUC of 1.00 on the present dataset, with a Micro-AUC score of 1.00 and a Kappa coefficient of 1.00. These results underscore the model’s ability to accurately classify TB infection status into three distinct categories: at least latent infection, BCG false positives, and non-infected individuals. The model’s performance is further supported by learning curve analysis, which indicates that it reaches optimal fitting with sample sizes between 150 and 650 cases, and maintains consistent accuracy when sample sizes exceed 800 cases. This highlights the model’s robustness and its potential for large-scale, high-altitude screening efforts, where the burden of tuberculosis is often underestimated due to challenging environmental conditions.

Although a formal health economic evaluation was beyond the scope of this study, the proposed “EC first, then PPD” algorithm has practical implications for resource-limited settings. The EC test is highly specific and can first definitively identify all infected individuals (Latent and Active). The subsequent PPD test, which is low-cost and widely available, is then only needed for the EC-negative group to further distinguish between BCG vaccination effects and true non-infection. This sequential approach could potentially reduce the number of unnecessary chest X-rays and further investigations compared to using either test alone or in parallel, optimizing the use of limited healthcare resources. Future studies should include a cost-effectiveness analysis to confirm this advantage.

The promising performance of the EC test and the combined model in our adolescent cohort suggests a valuable tool for TB screening in high-altitude schools. However, the performance of immune-based tests like the EC test can be influenced by age-related factors. As reviewed by Basu and Chakraborty (20), immune-based tests like EC may show reduced sensitivity in young children and immunocompromised individuals due to non-specific immune responses and the paucibacillary nature of pediatric TB.

This study has certain limitations. Firstly, the study population is predominantly composed of adolescents, and a recent study on differential diagnosis of active pulmonary TB suggests that age may influence the sensitivity and specificity of the EC skin test (21). Therefore, multi-center, large-sample studies in adult and elderly populations are necessary to evaluate the diagnostic performance across different age groups. Secondly, the small sample sizes in the at least latent infection group (*n* = 43) and the BCG-vaccinated group (*n* = 29), along with uneven distribution across categories, may have weakened the model’s ability to learn from minority categories. Future research should expand the sample size and consider refined weighting or data augmentation strategies. Thirdly, the study was limited to Seda County, a plateau pastoral area, and does not cover other high-burden regions with varying altitudes, climates, and BCG vaccination rates. Additionally, the lack of a health economics evaluation limits the generalizability of the findings. Future studies should validate the model in multi-center settings and conduct cost-effectiveness analyses. Fourthly, our diagnostic classification algorithm, while based on expert consensus, simplifies complex immunological states. It does not account for individuals who may have received BCG revaccination at age 7, those in whom the post-vaccination immune response has waned (leading to false-negative PPD results), or the possibility of true MTB infection in BCG-vaccinated individuals. Furthermore, potential allergic reactions or energy, which could lead to false-negative results, were not analyzed. The potential ‘booster effect’ of sequential skin testing was also not considered in this cross-sectional study design. Lastly, all participants in this study underwent both EC and PPD tests, which is not standard clinical practice and may lead to cross-reactivity or operational bias. Future studies should optimize testing protocols based on real-world clinical pathways to further improve the model’s practicality and scalability.

## 5 Conclusion

This high-altitude TB screening study demonstrated the EC skin test’s high sensitivity and specificity, supporting its utility in regions with high BCG vaccination rates. The combined EC + PPD decision tree model showed potential for precise classification of at least latent infections, BCG vaccinations, and uninfected individuals in this setting, exhibiting high performance on the study data. The model’s simple structure could facilitate field application, suggesting it is a promising tool for resource-limited high-altitude regions. Future multicenter studies are needed to verify its applicability across diverse populations and regions, and health economic evaluations are warranted to promote its widespread adoption in global high-burden areas.

## Data Availability

The original contributions presented in the study are included in the article/supplementary material, further inquiries can be directed to the corresponding author.
